# The relationship between smartphone addiction and aggression among Lebanese adolescents: the indirect effect of cognitive function

**DOI:** 10.1186/s12887-022-03808-y

**Published:** 2022-12-26

**Authors:** Feten Fekih-Romdhane, Diana Malaeb, Abir Sarray El Dine, Sahar Obeid, Souheil Hallit

**Affiliations:** 1 The Tunisian Center of Early Intervention in Psychosis, Department of psychiatry “Ibn Omrane”, Razi hospital, 2010 Manouba, Tunisia; 2 Faculty of Medicine of Tunis, Tunis El Manar University, Tunis, Tunisia; 3 School of Pharmacy, Lebanese International University, Beirut, Lebanon; 4 College of Pharmacy, Gulf Medical University, Ajman, United Arab Emirates; 5 Department of Biomedical Sciences, School of Arts and Sciences, Lebanese International University, Beirut, Lebanon; 6 Social and Education Sciences Department, School of Arts and Sciences, Lebanese American University, Jbeil, Lebanon; 7 School of Medicine and Medical Sciences, Holy Spirit University of Kaslik, P.O. Box 446, Jounieh, Lebanon; 8 Applied Science Research Center, Applied Science Private University, Amman, Jordan; 9 Research Department, Psychiatric Hospital of the Cross, Jal Eddib, Lebanon

**Keywords:** Smartphone addiction, Aggression, Cognitive function, Adolescence, Students

## Abstract

**Background:**

Despite a large body of research has shown that smartphone addiction (SA) is associated with aggressive behaviors, only a few mediators have been previously examined in this relationship among early adolescent students. No previous studies have explored, to our knowledge, the indirect role of cognitive function despite its great importance during this life period. This study is intended to verify whether cognitive function have indirect effects on the relationship between SA and aggression among high-school students in the context of Lebanese culture.

**Methods:**

This was a cross-sectional designed study, conducted between January and May 2022, and enrolling 379 Lebanese adolescent students (aged 13–17 years). The Cognitive Functioning Self-Assessment Scale, the Buss–Perry Aggression Questionnaire-Short Form, and the Smartphone Addiction Scale-Short form were used.

**Results:**

The bivariate analysis results revealed that higher SA and worse cognitive function were significantly associated with more physical aggression, verbal aggression, anger and hostility. The mediation analyses found that cognitive function mediated the association between SA and physical aggression, verbal aggression, anger and hostility. Higher SA was significantly associated with worse cognitive function and more physical aggression, verbal aggression, anger and hostility. Finally, worse cognitive function was significantly associated with more physical aggression, verbal aggression, anger and hostility.

**Conclusion:**

Our findings cautiously suggest that, to reduce adolescent students’ aggression, interventions that promote cognitive performance may be effective. Particularly, students who are addicted to smartphones and show aggressive tendencies require interventions designed to improve cognition function.

## Background

Over the last decades, the number of smartphone owners has been constantly increasing to reach 83.72% of the world’s population in 2022 (compared to 49.40% in 2016) [1], with the highest percentage of smartphone users being adolescent students (high school graduate or less) [1]. Smartphones are practical, and provide easy, convenient access to many services including unrestricted communication with others, academic materials access, and leisure online activities. In particular, smartphones have offered adolescents opportunity to develop their self-identity and personal autonomy, establish interpersonal relationships, be creative, and entertain [2, 3]. All these attractive attributes and its non-restricted use by space and time have led to the emergence of addictive smartphone behaviors, especially at a young age [4].

### SA among adolescent students

Adolescence is a critical period of heightened biological vulnerability to addiction, and of onset of addictive disorders [5, 6]. Previous studies investigating smartphone addiction (SA) using the most widely used measure (the Smartphone Addiction Scale-Short Version, SAS-SV) revealed high prevalence rates of SA worldwide among early adolescent students (e.g., 16.9% in Switzerland [7], 22.8% in China [8], 26.61% in Korea [9], 36.9% in Turkey [10], 37.1% in Iran [11], 42.9% in Brazil [12], 55.8% in Morocco [13], and 62.6% in the Philippines [14]). Using the same scale, we could found a study in Lebanon that surveyed young adults of the general population (aged 18 to 29 years), and found that 46.9% of participants had SA [15]. However, as far as we are aware, no prior studies have been interested in evaluating SA in Lebanese adolescent students.

Increased evidence supports the detrimental effects of SA that became significant and growing social and public health problems [16]. SA has been shown to negatively impact the students’ mental health, and to be linked to a variety of psychological problems including anxiety, depression, stress [17, 18], sleep problems [19], poor academic performance [20], peer relationship problems, self-harm and even suicidal ideation and behaviors [21, 22]. Another potential negative consequence that is gaining attention, due to its serious impacts on adolescents’ lives, is aggression [23]. Despite all these harmful effects, research related to this topic remains to date limited [24]. We join the view of Wilmer et al. who claimed that “it is crucial to understand how smartphone technology affects us so that we can take the steps necessary to mitigate the potential negative consequences” [25]; and we point to the necessity of deeply understanding how SA is related to poor socio-behavioral outcomes so that we can take the measures needed to overcome them.

### SA and aggression

According to Buss and Perry [26], aggression is classified into four dimensions : physical and verbal aggressions (i.e., instrumental component), hostility (i.e., cognitive component), and anger (i.e., affective component). Extensive research highlighted that aggressive behaviors, which refer to any observable act intended to inflict harm to others [27–29], are highly prevalent and represent an integral part of adolescents’ daily lives [30–32]. For instance, a large study from eight countries and 14,967 in-school adolescents aged 10–19 years revealed that 53.7% of participants exhibited interpersonal violence, among them 29.2% and 43.2% reported physical fighting and physical attacks, respectively [33]. Lebanese adolescents are more prone to engage in aggression given the environment saturated with violence in which they grow up and live [34]. A previous study among 568 Lebanese adolescents aged between 15 and 18 years revealed that 34.0% and 31.9% had moderate and high aggression respectively [35]. Indeed, the political instability, deteriorating economy and ongoing conflicts that Lebanon has known in the past years resulted in increased violence rates in schools and streets; that have gone so far as to be engaged in armed conflicts [34, 36].

Empirical studies have identified various risk factors of aggression in adolescence [37], mainly gender (boys display more physical aggression than girls) [38, 39], mental health disorders (most notably disruptive behavior disorders and attention-deficit/hyperactivity disorder (ADHD), alexithymia, anxiety and depression) [40, 41], family characteristics including single-parent household and divorced parents [42], and peer factors involving parental divorce [43], peer rejection, bullying, and loneliness [44]. Moreover, during adolescence, developmentally normative changes in social relationships, including decreasing parental supervision, increasing influence of peers and engaging in new risky behaviors (e.g. alcohol drinking, drug use and smoking) may also elevate risk for aggression [45, 46]. In addition, the adolescent brain evolves its capability to organize, regulate impulses, and weigh risks and rewards; however, these changes can make adolescents highly vulnerable to risk-taking behavior [47]. More particularly, studies showed that increased amygdala volume and decreased leftward asymmetry of the anterior cingulate cortex were associated with increased duration of aggressive behaviors during the interpersonal interactions [48].

A large body of correlational research has shown that SA is significantly related to aggressive behaviors. For example, positive correlations have been found between problematic cellular phone use and a number of behavioral problems, including aggression, in Taiwanese adolescent students [49]. Similarly, problematic smartphone use has been shown to be associated with aggression and hostility among young adults in Switzerland [50]. A Korean study by Um et al. showed that smartphone dependency (as assessed using a scale by Lee et al. [51]) significantly correlated with aggression among middle school students, suggesting that a “careful use of smartphones is necessary” in this population [23]. Another Korean study by Wee and Kang found that many forms of addiction (i.e., alcohol, gambling and SA) are significantly related to aggression [52]. A study by Khoo and Yang conducted among Singaporean students found that SA is a potential risk factor for the hostility facet of aggression [53]. In sum, most of the evidence came from Asian and Western countries, and supports a positive association between SA and aggression. According to Zarei [54], problematic smartphone use is one of the major variables affecting the aggressive behavior of students.

Aggression among early adolescents represents a serious problem that can significantly impede their development and lead to major clinical and social concerns, including school violence between peers [55], school drop-out [56, 57], substance abuse [57], physical violence and crime perpetration later in adulthood [56, 58, 59], as well as future economic difficulties and health problems [60]. This wide range of possible negative outcomes highlight that this topic deserves careful consideration at the scientific, clinical and policy levels.

### Cognitive function as a Mediator between SA and Aggression

Another substantial factor that can drive aggression among adolescents is cognitive functioning. It is well established that cognitive skills and functions are determinant in regulating adolescents’ thoughts and actions [61]. It is thus understandable that cognitive impairment poses a major risk of aggressive thoughts and behaviors. Heavy smartphone users would be highly prone to report cognitive failures during everyday life [62]. Some authors even suggested that the mere presence and/or the simple reminder of one’s smartphone could highly and adversely affect students’ cognitive functioning and performance [63].

On the other hand, cognitive impairment has been demonstrated as one of the negative consequences of SA [25, 64, 65]. Although research concerning the cognitive effects of smartphone use is still quite limited and longitudinal evidence is scant, a literature review by Wilmer et al. [25] showed that smartphones can be detrimental to a variety of cognitive domains, including mnemonic functioning, attentional capacities, and tendency to delay gratification. A more recent review by Liebherr et al. [64] found that smartphone use impacts working memory, inhibition, attention, among other cognitive functions. Regarding the student population in particular, a study from Singapore found that smartphone overuse impaired students’ cognitive abilities (i.e., executive functions) [66]. In Turkey, SA has been found to negatively affect students’ cognitive flexibility [67].

Given that both SA and cognitive function are involved in aggression, we suggest that cognitive function could play an indirect role in fostering the relationship between SA and aggression. Investigating the cognitive function effects could provide valuable information about how SA can affect early adolescent students’ brain and behaviors during a period of increased developmental plasticity. Only a few mediators have been previously examined in the relationship between SA and aggression among early adolescent students (e.g., peer attachment, ego-resilience, parenting behavior; [23]); however, to our knowledge no studies have explored the mediating role of cognitive function despite its great importance during this life period.

### The present research

To date, there is little amount of research focused on the relation between smartphone use and its subsequent socio-behavioral outcomes [53]. Khoo and Yang [53] recently suggested that, among the various aspects of smartphone use, SA in particular is potentially impactful to students’ aggression risk, and thus requires more research and targeted interventions. We decided to perform this study for several reasons. First, although an increasing number of studies supported the notion that SA could predict adolescents’ aggression, only a few studies have attempted to test the mediating effects of personal factors in the association between SA and aggressive behaviors, which has substantially restrained the development of interventions [68]. Second, prior research examining the relation SA and aggression involved children, primary school students [69, 70], or young-adult university students [71]; whereas, there are a few or no studies conducted among early-adolescent high school students despite being particularly vulnerable to develop both addictive and aggressive behaviors with long-lasting consequences [72, 73]. Third, as previously said, the vast majority of studies on this topic emerged from Asia and the developed world, with no studies from the low-middle-income countries of the Middle East and North Africa region. Given that the findings related to both SA [71] and aggression [74] might vary cross-culturally, we believe that the present study has an original value and contributes to the literature by adding data from an unexplored country and region. Based on these gaps identified in the existing literature, our study is intended to verify whether cognitive functions have indirect effects on the relationship between SA and aggression among high-school students in the context of Lebanese culture.

## Methods

### Study design and Procedure

This was a cross-sectional designed study, conducted between January and May 2022, and enrolling 379 adolescent students currently residing in Lebanon (13 to 17 years old), from all Lebanese governorates (Beirut, Mount Lebanon, North, South, and Bekaa). Our sample was chosen using the snowball technique; a soft copy of the questionnaire was created using google forms software, and an online approach was conceived to proceed with the data collection. The study’s main aims and goals, in addition to instructions for filling the questionnaire, were conveyed online for the participants, prior to their participation. Later, initial participants approached by the research team were asked to recruit other participants they know, preferably as diverse as possible with regard to place of habitat within the Lebanese governorates and within the same age interval required to participate in the study. Internet protocol (IP) addresses were examined to ensure that no participant took the survey more than once. There were no credits received for participation. Included were Lebanese adolescents, aged between 13 and 17 years and who have a smartphone. Excluded were those who do not fulfill one of these criteria.

### Minimal sample size calculation

A minimal sample of 127 was deemed necessary using the formula suggested by Fritz and MacKinnon [75] to estimate the sample size: $n=\frac{L}{f2}+k+1$, where f=0.26 for a small to moderate effect size, L=7.85 for an α error of 5% and power β = 80%, and 10 variables to be entered in the model.

### Questionnaire

The first part of the questionnaire included an explanation of the study topic and objective, a statement ensuring the anonymity of respondents and an explanation for the student to get his/her parents’ approval before participation. The student had to select the option stating “I got my parents’ approval and consent to participate in this study” to be directed to the questionnaire.

The second part of the questionnaire contained sociodemographic information about the participants (age, gender, governorate, current self-report weight and height). The Body Mass Index (BMI) was consequently calculated as per the World Health Organization [76]. The household crowding index, reflecting the socioeconomic status of the family [77], is the ratio of the number of persons living in the house over the number of rooms in it (excluding the kitchen and the bathrooms). The physical activity index is the cross result of the intensity, duration, and frequency of daily activity [78]. Regarding the financial burden, respondents were asked to answer the question “How much pressure do you feel with regard to your personal financial situation in general?” on a scale from 1 to 10, with 10 referring to overwhelming pressure.

The third part included the scales used in this study:

#### The Buss–Perry Aggression Questionnaire-Short Form (BPAQ-SF)

Validated in Lebanon [79], the Buss-Perry Aggression Questionnaire-Short Form (BPAQ-SF) [80] is a short version of the BPAQ and consists of 12 Likert-type items rated on a 5-point ordinal scale and organized into four scales of three items each: Physical Aggression, Verbal Aggression, Anger, and Hostility. Bryant and Smith (2001) decided to change the original 5-point scale to a 6-point scale to eliminate the scale’s midpoint and force respondents to decide whether each statement was characteristic of them. Higher scores indicate higher levels of aggression. The Cronbach’s alpha values were as follows: physical aggression (α = 0.66), verbal aggression (α = 0.55), hostility (α = 0.72) and anger (α = 0.71).

##### Smartphone addiction scale-short version (SAS-SV)

The SAS, validated in Lebanon [81], is a ten-item scale used to evaluate SA among adolescents [82]. The total score was computed by adding the answers of these 10 items, with higher scores reflecting higher SA (Cronbach’s alpha = 0.90).

#### Cognitive Functioning Self-Assessment Scale (CFSS)

The questionnaire included 18 statements; participants were required to estimate, on a five-point scale anchored ‘‘never-always’’, the frequency of each described situation in the past 12 months (e.g. *Difficulty in performing two tasks simultaneously; Difficulty in performing mental calculatio*n) [83] (Cronbach’s alpha = 0.95). Higher scores indicate worse cognitive function.

## Translation procedure

The forward and backward translation method was applied to different scales. The English version was translated to Arabic by a Lebanese translator who was completely unrelated to the study. Afterwards, a Lebanese psychologist with a full working proficiency in English, translated the Arabic version back to English. The initial and translated English versions were compared to detect and later eliminate any inconsistencies.

### Statistical analysis

SPSS software version 23 was used to conduct data analysis. Cronbach’s alpha values were computed for each scale. We had no missing data since all questions were required in the Google form. All aggression subscales scores were normally distributed, with its skewness and kurtosis varying between − 1 and + 1 [84]. The Student t and ANOVA tests were used to compare two and three or more means respectively, whereas the Pearson correlation test was used to compare two continuous variables. The PROCESS SPSS Macro version 3.4, model four [85] was used to calculate three pathways. Pathway A determined the regression coefficient for the effect of smartphone addiction on cognitive function; Pathway B examined the association between cognitive function and aggression, and Pathway C’ estimated the direct effect of smartphone addiction on aggression. An indirect effect was deemed significant if the bootstrapped 95% confidence intervals of the indirect pathway AB did not pass by zero. Variables that showed a *p* < 0.25 in the bivariate analysis were entered in the multivariable and mediation models. Significance was set at a *p* < 0.05.

## Results

### Sociodemographic and other characteristics of the participants

A total of 379 adolescents participated in this study; their mean age was 16.07 ± 1.19 years, with 64.9% females. Other characteristics are summarized in Table 1. The results showed that 157 (41.4%) adolescents had smartphone addiction, 49 (36.8%) boys (scores ≥31) and 108 (43.9%) girls (scores ≥33).

**Table 1:** Sociodemographic and other characteristics of the participants (*N* = 379)

Variable	N (%)
**Sex**	
Male	133 (35.1%)
Female	246 (64.9%)
	Mean ± SD
** Age (in years)**	16.07 ± 1.19
** Physical activity index**	27.78 ± 20.15
** Household crowding index (persons/room)**	1.26 ± 0.74
** Body Mass Index (kg/m**^**2**^ **)**	22.33 ± 3.79
** Financial burden**	4.96 ± 2.80
** Physical aggression**	6.59 ± 2.90
** Verbal aggression**	7.46 ± 2.85
** Anger**	8.12 ± 3.29
** Hostility**	6.80 ± 3.12
** Cognitive function**	25.27 ± 14.22
** Smartphone addiction**	30.34 ± 11.36

### Bivariate analysis

The bivariate analysis results are shown in Tables 2 and 3. A higher mean physical aggression score was seen in males compared to females (7.03 vs. 6.36; *p* = 0.043), whereas a higher mean anger score was seen in females compared to males (8.45 vs. 7.53; *p* = 0.009). Higher SA and worse cognitive function were significantly associated with more physical aggression, verbal aggression, anger and hostility. Older age was significantly associated with more verbal aggression. Higher BMI was significantly associated with more physical aggression, whereas more financial burden was significantly associated with more hostility.

**Table 2:** Bivariate analysis of the categorical variables associated with the aggression scores

Variable	Physical aggression	Verbal aggression	Anger	Hostility
**Sex**				
Male	7.03 ± 3.29	7.50 ± 3.04	7.53 ± 3.22	6.55 ± 3.25
Female	6.36 ± 2.64	7.44 ± 2.76	8.45 ± 3.29	6.94 ± 3.04
*P*	**0.043**	0.842	**0.009**	0.251

Numbers in bold indicate significant *p*-values

**Table 3:** Bivariate analysis of the continuous variables associated with the aggression scores

Variable	Physical aggression		Verbal aggression		Anger		Hostility	
	r	*P*	r	*p*	r	*p*	r	*p*
Physical aggression	1	-						
Verbal aggression	0.46	**< 0.001**	1	-				
Anger	0.50	**< 0.001**	0.51	**< 0.001**	1	-		
Hostility	0.49	**< 0.001**	0.49	**< 0.001**	0.67	**< 0.001**	1	-
Cognitive function	0.23	**< 0.001**	0.27	**< 0.001**	0.36	**< 0.001**	0.49	**< 0.001**
Smartphone addiction	0.18	**< 0.001**	0.29	**< 0.001**	0.31	**< 0.001**	0.33	**< 0.001**
Age	0.02	0.748	0.13	**0.014**	0.08	0.145	0.02	0.645
Physical activity index	0.03	0.547	− 0.01	0.808	− 0.04	0.442	− 0.06	0.213
Household crowding index	− 0.05	0.344	− 0.07	0.172	0.04	0.475	− 0.05	0.332
Body Mass Index	0.10	**0.043**	0.07	0.154	0.03	0.604	0.07	0.181
Financial burden	0.07	0.149	0.05	0.349	0.09	0.086	0.14	**0.006**

Numbers in bold indicate significant *p*-values, *r*  Pearson correlation coefficient. Higher cognitive function scores indicate worse cognitive function

### Indirect effect analysis

**Fig. 1:**
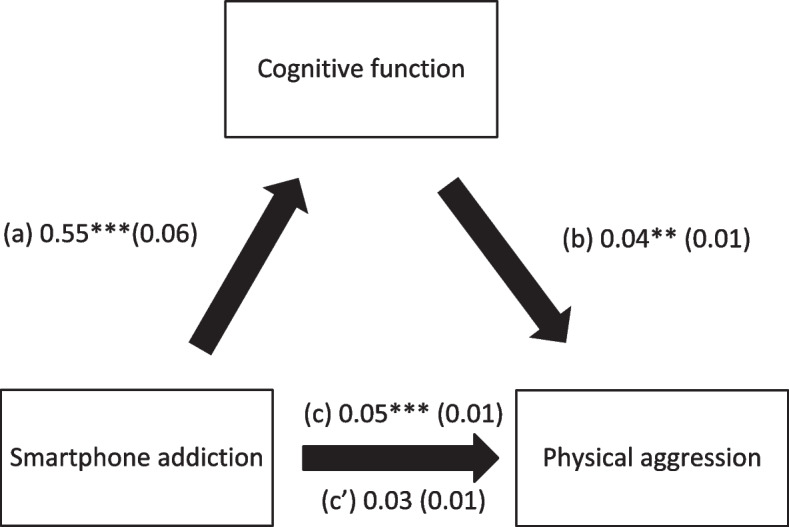
(**a**) Relation between smartphone addiction and cognitive function (R^2^ = 25.56%); (**b**) Relation between cognitive function and physical aggression (R^2^ = 8.13%); (**c**) Total effect of the relation between smartphone addiction and physical aggression (R^2^ = 5.54%); (c’) Direct effect of the relation between smartphone addiction and physical aggression. Numbers are displayed as regression coefficients (standard error). ***p < 0.001; **p < 0.01

**Fig. 2:**
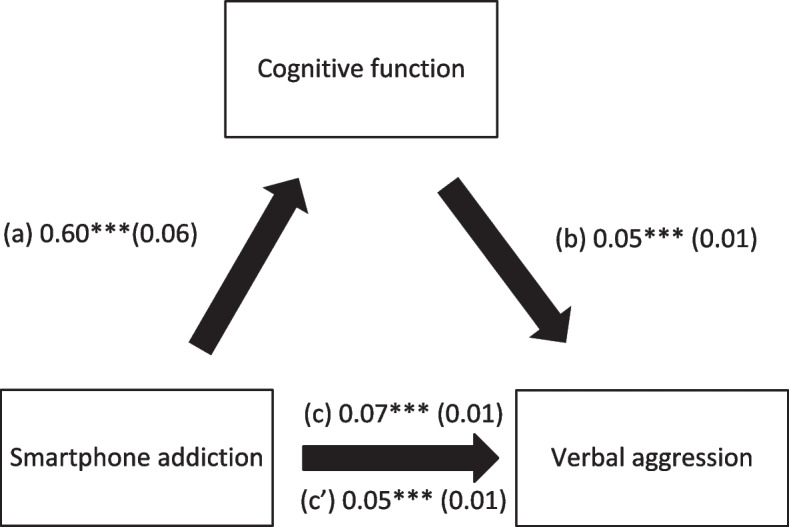
(**a**) Relation between smartphone addiction and cognitive function (R^2^ = 23.64%); (**b**) Relation between cognitive function and verbal aggression (R^2^ = 12.29%); (**c**) Total effect of the relation between smartphone addiction and verbal aggression (R^2^ = 10.33%); (c’) Direct effect of the relation between smartphone addiction and verbal aggression. Numbers are displayed as regression coefficients (standard error). ***p < 0.001

**Fig. 3:**
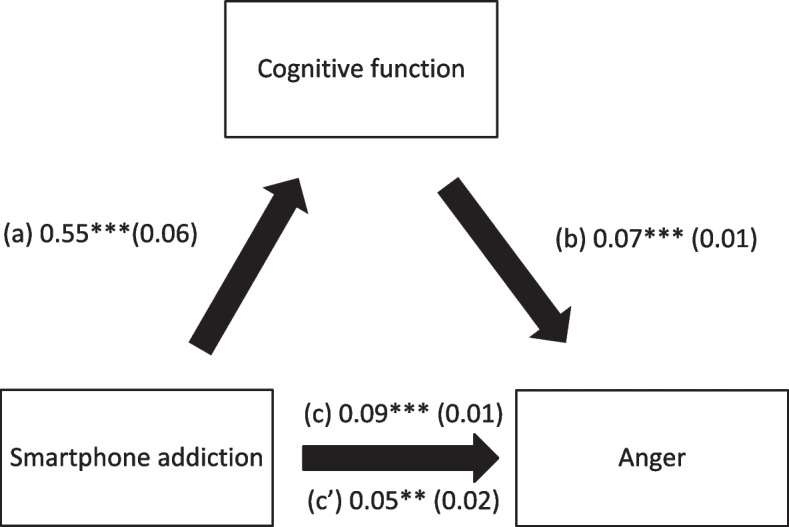
(**a**) Relation between smartphone addiction and cognitive function (R^2^ = 25.52%); (**b**) Relation between cognitive function and anger (R^2^ = 16.64%); (**c**) Total effect of the relation between smartphone addiction and anger (R^2^ = 10.76%); (c’) Direct effect of the relation between smartphone addiction and anger. Numbers are displayed as regression coefficients (standard error). ***p < 0.001; **p < 0.01

**Fig. 4:**
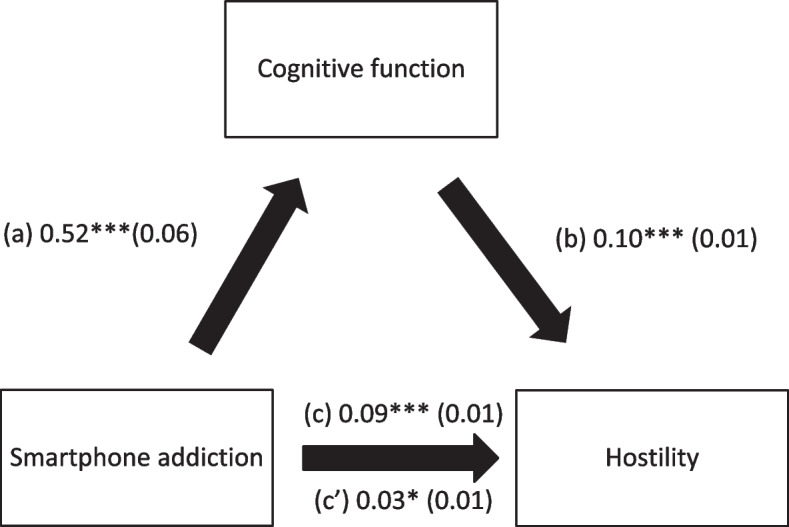
(**a**) Relation between smartphone addiction and cognitive function (R^2^ = 27.19%); (**b**) Relation between cognitive function and hostility (R^2^ = 25.61%); (**c**) Total effect of the relation between smartphone addiction and hostility (R^2^ = 11.23%); (c’) Direct effect of the relation between smartphone addiction and hostility. Numbers are displayed as regression coefficients (standard error). ***p < 0.001; **p < 0.01; *p < 0.05

Cognitive function mediated the association between SA and physical aggression, verbal aggression, anger and hostility (Table 4; Figs. 1, 2, 3 and 4). Higher SA was significantly associated with worse cognitive function and more physical aggression, verbal aggression, anger and hostility. Finally, worse cognitive function was significantly associated with more physical aggression, verbal aggression, anger and hostility.

**Table 4:** Indirect effect analyses results, taking smartphone addiction as the independent variable, cognitive function as the mediator and the aggression scores as dependent variables

	Direct effect			Indirect effect		
	Beta	SE	*p*	Beta	Boot SE	Boot CI
Physical aggression	0.03	0.01	0.073	0.02	0.01	0.01-0.04*
Verbal aggression	0.09	0.03	< 0.001	0.03	0.01	0.01-0.06*
Anger	0.08	0.03	0.006	0.06	0.02	0.03-0.09*
Hostility	0.15	0.02	< 0.001	0.06	0.02	0.03-0.09*

* indicates significant indirect effect

## Discussion

Lebanon is a young society in which 44% of people are under the age of 24 [36, 86]. Being at this developmental stage carries a risk of unhealthy and risky behaviors, such as SA and aggression. Indeed, previous studies revealed high rates of both SA and aggression in Lebanese youth [15, 34], highlighting the need to investigate the relationship between these two entities in this specific population and context, to help improve development and implementation of socially and culturally tailored prevention and intervention approaches. In this study, we tested the hypothesis that cognitive functions mediate the relationship between SA and the four aggression dimensions among Lebanese high-school students. For this, we established path analyses models where SA was taken as an independent variable and each of aggression dimensions as dependent variables. All models showed partial mediation, confirming our hypothesis.

As for the direct effects of SA on aggression, our findings were in line with the existing literature. There is some evidence that SA significantly and positively contributes to aggressive tendencies in students [54, 87, 88]. Previous studies from different countries (e.g., Korea [23], Taiwan [89], Singapore [53], Switzerland [50]) have shown that students who excessively use a smartphone are prone to heightened aggression. Other studies also found that specific online activities are linked to more aggression among students, such as smartphone gaming [90] and online gambling [91]. Various theories have been advanced to explain the relationship SA-aggression. For example, it has been suggested that, because students are under high levels of stress, the overuse of smartphones may easily trigger chronic fatigue and mental health problems; that, in turn, lead to a loss of self-control in challenging situations [92]. In the same line, a prospective study revealed that students’ high lack of self-control predicted aggressive behaviors [93]. Another possible explanation is that students with SA are heavily exposed to violent and suggestive applications; which may result in a loss of social skills and coping abilities [94]. A recent cross-sectional study conducted in Singapore showed that students’ addictive smartphone use predicted the cognitive component of aggression (i.e. hostility) [53]. Authors explained their results by the fact that SA might generate hostile cognitive beliefs, such as high levels of jealousy or suspiciousness [95]. In addition, SA is highly disruptive leading to heightened negative affect, which in turn triggers aggression [53].

Although we found evidence supporting that SA is associated with aggression, we cannot establish the causality or directionality of the observed relationship. Some previous research rather supported the path leading from aggression to SA [68]. It has been suggested that adolescents with aggressive tendencies may turn to their smartphones to better express various urges and pressures in a space where internal aggressiveness can be easily and conveniently expressed [17]. Also, aggressive adolescents may excessively use their smartphones because they experience social difficulties, such as poor peer relationships [96]. These data along with our findings suggest a bidirectional relationship between SA and aggression, and call for further longitudinal research using different timeframes.

Regarding the indirect effects, we found that higher SA was significantly and inversely associated with cognitive function; and that cognitive function was negatively associated with aggression (all dimensions). These findings are in line with prior longitudinal evidence that has identified the role of cognitive deficits in the development of later adolescents’ aggression [97]; as well as the role of smartphone use in decreasing cognitive abilities [98]. In addition, our expectations could be confirmed, showing that cognitive functions partially mediate the relationship between SA and all aggression components. Different theoretical explanations could be proposed for these findings. First, many previous studies demonstrated that SA negatively impacts cognitive functions (for review, see [25, 64, 65]). For instance, individuals with SA or those who use their smartphones in situations where it is dangerous or prohibited more often show low trait inhibitory control [99, 100]. This lack of self-control has also been robustly associated with aggression [101]. Second, both smartphone addiction [102, 103] and deficits in cognitive functions [104, 105] are linked to higher levels of negative affect; which may in turn lead to more aggressive behaviors among adolescents [106, 107]. Third, SA exposes to important structural and functional brain changes, including white matter changes in brain regions involved in emotional processing and executive functions [108]. At the same time, white matter abnormalities have been suggested to potentiate aggressive tendencies in non-clinical adolescents [109].

### Study strengths & limitations

The present study has strengths that deserve to be mentioned. First, this topic has not received previous scrutiny in low-middle income countries with an Arab cultural background. In addition, this study is innovative in examining cognitive functions as a mediator in the relationship SA-aggression; this has not yet been actively researched. Another strength lies in considering the multifaceted construct of aggression with four components (i.e., physical and verbal aggression, anger, hostility) [26, 95], while most of the previous research considered aggression as a unidimensional construct [110], or only treated one aspect of aggression (e.g., anger, [111].

This study has also some limitations to be noted and that point to suggestions for future research. First, the cross-sectional design precludes any causal inferences. Further longitudinal research is needed to further ascertain the directionality of the investigated relationships. Second, the use of self-reported measures might have led to recall bias or social desirability issues; and calls for the use of objective measures in future studies [112]. Third, we only examined SA, whereas smartphone-related behaviors are complex and multidimensional. Thus, examining the various activities, contents and patterns of smartphone use in additional studies would be useful [113].

### Clinical, research and policy implications

Today’s students have been exposed to smartphones from a very young age, are particularly vulnerable to SA because of their age-related characteristics (including a less-developed self-control [92, 114, 115], and are not necessarily aware of the smartphones’ potential harmful impacts on their development, mental health and well-being. There is enough evidence to suggest that SA leads to aggression in adolescent students [54, 87, 88]. Our findings provide further support to these data, and could help guide targeted prevention and intervention strategies for aggression in adolescent students. Despite aggression is occurring at staggering rates in Lebanon, there are no programs so far to monitor students’ aggressive and violent behaviors. Therefore, in light of our findings and prior evidence, we highlight the urgent and basic need to implement school programs that target in-school adolescents, especially those who show addictive smartphone use behaviors, to combat aggression and violence in school settings. We recommend that one way to overcome aggression efficiently could be sensitizing students on the potential harms of SA and helping them to monitor their duration and frequency of smartphone use [54]. Furthermore, specific interventions designed to reduce reactive aggression such as cognitive reappraisal, self-control training, cognitive control training, and Mindfulness could correspondingly be instigated in schools settings [116]. In addition, four strategic priorities could be recommended: (1) establishing recreational services which encourage students to engage in other leisure activities than their smartphone; (2) developing and implementing various educational programs which raise awareness about smartphone addiction among students; (3) developing policies and guidelines limiting the usage of smartphones during lectures; (4) establishing free and accessible sports facilities in all schools. Moreover, schools could implement a smartphone-based coaching program for addiction prevention among students [117]; the latter consists on an individually tailored intervention approach effective in increasing life skills and reducing risk behaviors in a group of adolescents with a particular high risk of addictive behaviors [118].

Another important finding of this study that might offer a potential avenue for intervention, relates to the indirect role of cognitive function in the relation SA-aggression. This implies that, to reduce adolescents’ aggression, interventions that promote cognitive performance may be effective. These interventions include activities such as problem-solving training, mnemonic training, and guided imagery [119]. Particularly, students who are addicted to smartphones and show aggressive tendencies require interventions designed to improve cognitive function. In other words, we suggest that moderating the cognitive function may decrease the effect of SA on aggression. Future longitudinal and experimental research is required to better understand the interactions between SA and aggression, and ascertain the indirect effects of cognitive function in this relationship. Future research need to consider the multidimensionality of smartphone use [53, 111], aggression [26, 95], and cognitive function [120].

## Conclusion

This study provides empirical evidence to test a mediation model exploring whether cognitive function underlies the relationship between SA and aggression. The findings can help educators, researchers, policy makers, and school counselors advance knowledge on this critical issue among students, and contribute to the development of effective prevention and intervention strategies. The main practical implications are that students should also be educated about the direct and indirect negative effects of SA, including the occupation of cognitive capacities and a heightened aggression. Promoting healthy ways of using smartphones could be one of the potential and effective strategies to prevent aggression in adolescent students. Taking measures to decrease the level of smartphone addiction and improve cognitive function may be effective in reducing students’ aggressive behaviors. Future research is needed to confirm our findings and help develop strategies for prevention and intervention.

## Data Availability

All data generated or analyzed during this study are not publicly available as per the ethics committee policies. The dataset supporting the conclusions is available upon request to the corresponding author (SH).
